# A Case Series and Literature Review of Craniofacial Fibrous Dysplasia

**DOI:** 10.7759/cureus.56771

**Published:** 2024-03-23

**Authors:** Padmashri P Kalmegh, Alka Hande

**Affiliations:** 1 Department of Oral and Maxillofacial Pathology, Sharad Pawar Dental College and Hospital, Datta Meghe Institute of Higher Education and Research, Wardha, IND; 2 Department of Oral Pathology and Microbiology, Sharad Pawar Dental College and Hospital, Datta Meghe Institute of Higher Education and Research, Wardha, IND

**Keywords:** monostotic, craniofacial, gnas1 mutation, benign fibro-osseous lesions, fibrous dysplasia

## Abstract

Craniofacial fibro-osseous lesions (CFOLs) are a diverse group of relatively rare entities whose etiology ranges from reactive to dysplastic with a potential for malignant transformation. It is distinguished by the replacement of bone with fibrous tissue, that subsequently develops different degrees of calcification. Fibrous dysplasia (FD) is a component of the fibro-osseous lesion spectrum. The clinical spectrum of FD is wide, ranging from minor monostotic lesions affecting a single bone to devastating polyostotic disease involving the entire skeleton. FD produces asymmetry, which impairs face aesthetics. FD leads to bone differentiation, disintegration, and disorganization. It depicts a cellular collagenous stroma lacking mitotic figures and pleomorphism. Blood capillaries are evenly distributed, as are elongated trabeculae of woven or lamellar bone with uneven curves (often referred to as the Chinese letters pattern). Three types of FD patterns can be identified by computed tomography (CT) imaging: a cystic pattern, a homogeneously dense pattern, and a ground-glass pattern. The cornerstone of treatment is surgery, although the method varies depending on the location, size, and symptoms of the lesion. As an alternative to surgery, the use of bisphosphonates to reduce osteoclastic activity is under consideration. In this case series, we present three cases of FD involving the maxilla and mandible. We aim to correlate the clinical presentation, histological features, and radiographic findings, to promote early diagnosis, treatment, and better prognosis of the patient.

## Introduction

Fibrous dysplasia (FD) is a not inheritable familial condition in which the normal structure of bone has been replaced by immature, randomly dispersed fibro-osseous tissue, causing deformity, fractures, discomfort, and impairment in function [1]. It has been defined as a neoplastic hamartomatous condition of bone maturation and remodelling [2]. FD accounts for 2.5% of all bone tumours and 7% of all benign bone tumours, that are caused by congenital, metabolic, and genetic abnormalities [3]. It is considered an unusual developmental abnormality with three types: monostotic FD (MFD), polyostotic FD (PFD), and craniofacial. The literature describes a PFD with skin lesions that are pigmented, and hormonal dysfunctions such as earlier puberty in females and hyperthyroidism. There is also a craniofacial variant of FD that is limited to the bones of the craniofacial complex such as the sphenoid and occipital [2]. The fibro-osseous anomaly in FD causes disordered and disorganized structural characteristics. Because FD is uncommon and often goes misdiagnosed, its epidemiology is still poorly known [4]. The aetiology is linked to a mutation in the alpha-subunit (Gs-alpha) of the G signalling coupling protein expressed by gene GNAS (guanine nucleotide binding protein alpha stimulating) [5]. The disease has been categorized as quiescent, nonaggressive or aggressive based on their clinical characteristics [6]. Bone pain and recurrent fractures are the most prevalent manifestations during childhood and adolescence, subsequently followed by malformation and neurologic compression, particularly when the skull and face bones are affected [3]. Malignant transformation occurs in 0.4% to 4% of cases [6]. FD is diagnosed based on clinical, radiographic, and histological findings [7]. However, the main objective of therapy is always to maintain function, the enhancement of aesthetics by treating the fundamental defect is also a legitimate purpose [1]. Here, we present a case series and insights into the etiologic, clinical, and histological aspects and possible pathogenic pathways of FD.

## Case presentation

**Figure 1:**
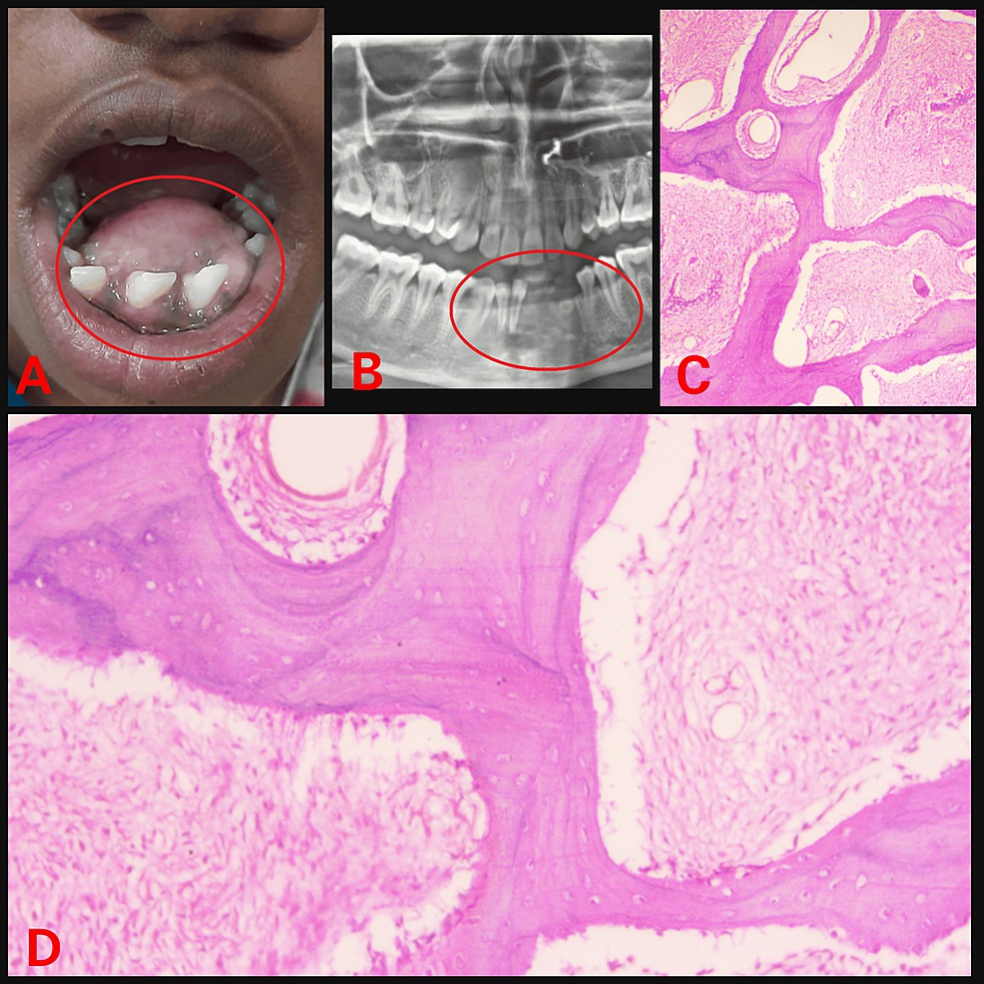
(A) Intraoral swelling; (B) Orthopantomogram showing radiolucency; (C) Haematoxylin and eosin (H&E) stained section under low power view; (D) H&E stained section under high power view.

We report a series of three cases of monostotic fibrous. The first patient had swelling present on the anterior mandibular region extending lingually from 35 to 45. She was alright one year back then started experiencing swelling in her lower front region that was initially small (Figure 1).

**Figure 2:**
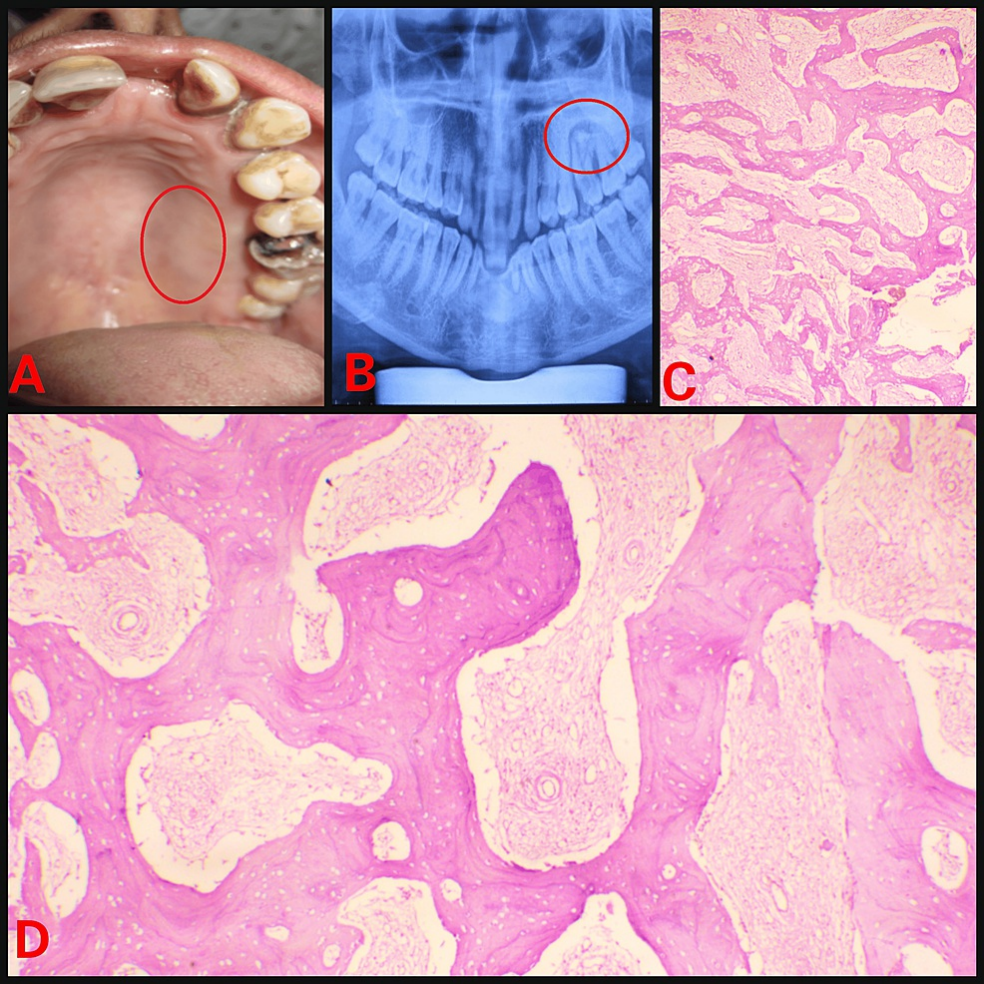
(A) Intraoral swelling; (B) Orthopantomogram showing radiolucency; (C) Haematoxylin and eosin (H&E) stained section under low power view; (D) H&E stained section under high power view.

Because of swelling, there is spacing in the lower anterior teeth which hampers aesthetics. The patient reported difficulty in chewing and talking. The second patient had swelling present on the left side of the maxilla extending on the palatal aspect from 24 to the distal of 27 with no extraoral asymmetry (Figure 2).

**Figure 3:**
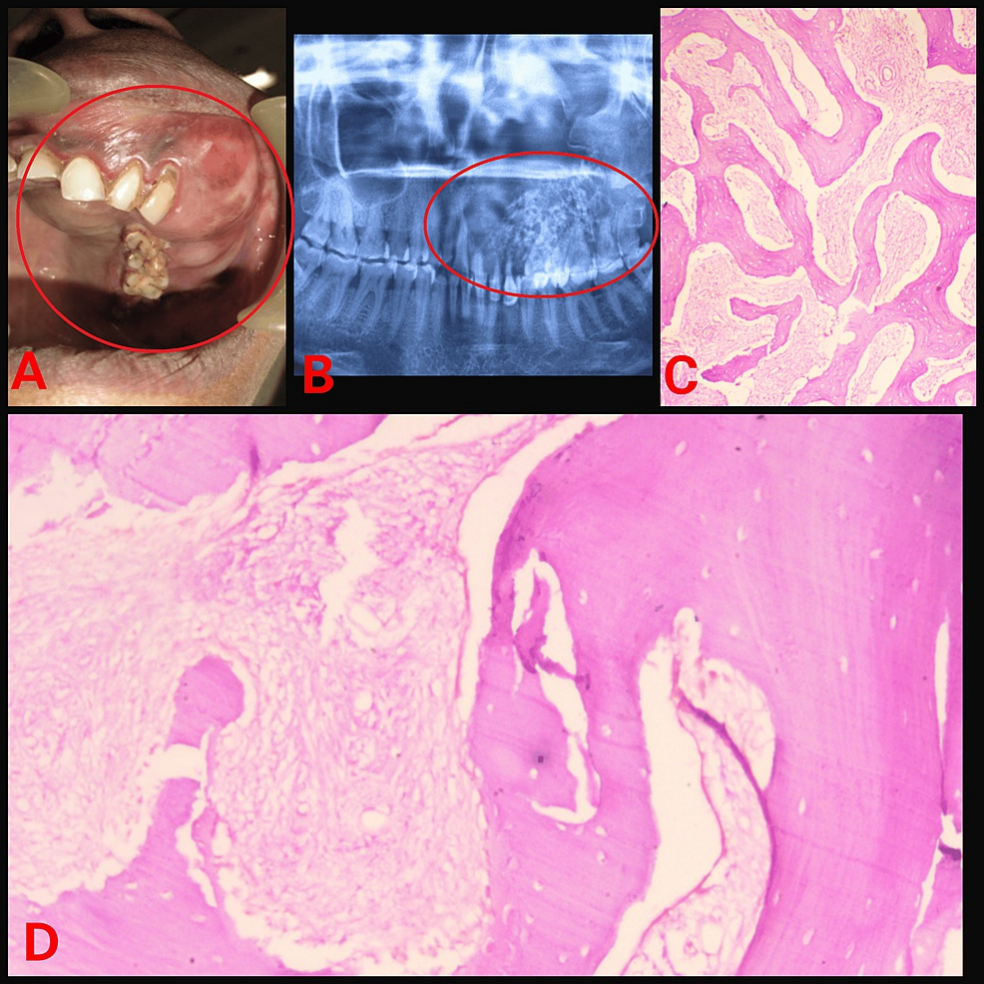
(A) Intraoral swelling; (B) Orthopantomogram showing radiolucency; (C) Haematoxylin and eosin (H&E) stained section under low power view; (D) H&E stained section under high power view.

The third patient came with a complaint of swelling present on the left side of the maxilla extending on the buccal as well as the palatal aspect from 11 to 28 showing facial asymmetry on the affected side (Figure 3).

The patient reported that initially, the swelling was present on the buccal aspect only but gradually it involved the palatal region. On examination marked facial asymmetry was evident compromising the aesthetics. Radiographic and haematological examinations were carried out for all patients. In case 1, surgical excision followed by bisphosphonate therapy was advocated. On follow-up evaluation, the patient does not report any complaints. In case 2, looking at the small size of the lesion, the patient was under regular observation for 15 days for three months. In case 3, radical excision with reconstruction and conservative bone shaving of the lesion was done. We advised biopsy to all patients for further histopathological evaluation to rule out the diagnosis. The demographic details, microscopic features, radiographic findings, and laboratory investigations of all cases are depicted in Table 1.

**Table 1:** Demographic details, microscopic features, radiographic findings and laboratory investigations M - Male, F - Female, U/L - units per liter

Sr. No.	Age/Sex	Area of chief complaint	Size	Radiographic features	Histopathologic features	Alkaline phosphatase level (Normal range: 30-130 U/L)
Case 1 (Figure 1)	20/F	Lower front region	2.5 x 2 cm	Radioopaque lesion	A lesion in the immature phase: Osteocytes are large and collagen fibers of trabeculae are seen extending out into fibrous tissue. Wide osteoids seen in some places. Connective tissue stroma composed of blood vessels and fibroblasts. Osteoblastic rimming is absent.	288 U/L
Case 2 (Figure 2)	28/M	Upper left posterior alveolus	1.2 x 0.8 cm	Radiolucent lesion	A lesion in the immature phase: Irregular and curvilinear trabeculae scattered throughout the lesion without any definite arrangement pattern of arrangement. Osteoblastic rimming is absent.	208 U/L
Case 3 (Figure 3)	58/F	Upper left posterior alveolus	3 x 4 cm	Radiolucent lesion	Lesion in mature phase: Presence of lamellae along with hematoxyphilic resting lines is appreciated. Osteoblastic rimming is absent.	305 U/L

## Discussion

Benign fibro-osseous lesions (BFOLs) encompass a wide range of bony abnormalities, comprising reactive, developmental, and dysplastic processes. These are pathological conditions characterised by the replacement of normal bone with distinct amounts of fibrous and mineralized tissue [8]. There are additional fibro-osseous entities, such as ossifying fibroma and cemento-osseous dysplasia [2]. FD is a benign intraosseous condition in which the medullary bone is replaced by fibrous connective tissue, resulting in underdeveloped and insufficiently calcified bone [7]. In 1938, Lichtenstein first coined the term FD [2]. Three distinct clinical forms of FD are: MFD affecting 70-80% of cases involving single bone; PFD affecting 20-30% of multiple bones; and the McCune-Albright syndrome, which occurs when the polyostotic form is accompanied by cutaneous and endocrine findings [9].

FD accounts for around 7% of benign bone diseases and 2.5% of all bone lesions [6]. It affects approximately 1:30,000 individuals [10]. The majority of cases of FD occur in the early or second decades of life, showing female prevalence 2:1 and typically having minimal progression and no symptoms [7]. If it is assumed that all FDs develop in childhood or adolescence, the majority of monostotic instances go undiagnosed until they become active or reactivate later in life, at the point where they are diagnosed for the first time [10]. Despite the fact that precocious puberty is most common, 21% of affected cases exhibit excessive growth hormone release [9]. However numerous authors, including Waldron et al., believe that the majority of cases "burn out" in early adulthood when skeletal maturity is attained. Eisenberg and Eisenbud believe that there are no long-term investigations of FD cases to back up this claim. Because many cases of FD are painless, it is entirely possible to diagnose a long-standing lesion in the advancing stage. The most frequently affected craniofacial bone is the maxilla (in 58% of cases) followed by the “mandible (43%)”, “frontal (33%)”, “sphenoidal (29%)”, “ethmoidal (24%)”, “parietal (14%)”, “temporal (5%)”, and “occipital” bones (3-4%) [11].

The pathophysiology of FD could be because of the varied behaviour and presentation [1]. It is caused by a deficiency affecting undifferentiated pluripotent stem cells. During the histodifferentiation phase of the embryo, a genetic mutation or deletion occurs in the gene encoding an intracytoplasmic transducer protein, GNAS1, which is necessary for bone formation. Any daughter cell of this malformed pluripotent stem cell that develops after birth is capable of generating disorganized, fibrous bone instead of developed bone [12]. Mutations can occur at one of two points: “Arginine (Arg201)” (> 95% cases) [13,14]. The mutation causes impaired intrinsic guanosine triphosphate (GTPase) activity of Gsα, leading to improper cyclic adenosine monophosphate (cAMP) mediated signalling. Excess cAMP production is therefore observed in the mutant cells [15]. Because of the increased cAMP, bone marrow stromal cells (BMSCs) are formed, but they are unable to develop into osteoblasts, adipocytes, or cells that contribute to haematopoiesis [1].

Immature osteoprogenitors proliferate due to inadequate differentiation of bone-forming precursors into mature osteoblasts and osteocytes, resulting in an excess of aberrant bone matrix, primarily composed of woven bone. Mutant osteoblastic cells overexpress receptor activators of nuclear factor kappa-B ligand and interleukin-6, which activates osteoclasts. This increases bone resorption and replaces bone with an excess of disordered collagenous matrix, causing the FD lesion to enlarge [16,17]. In addition, the conventional classifications of FD into MFD, PFD and McCune-Albright types might be a reflection of the timing of mutation and consequently, the initial bulk of FD precursor cells. While the MFD may emerge after birth, the PFD may emerge during fetal life. This is consistent with evidence demonstrating that the polyostotic form does not originate from the monostotic form [11].

Among those diagnosed with craniofacial FD, McCune-Albright syndrome accounted for 7%, PFD for 47%, and MFD for 56% of cases [18]. MFD predominantly involves maxilla. Long bone involvement is prevalent in PFD, and symptoms include pain, deformity, and pathological fracture [2]. MFD is ten times more prevalent than PFD, having a unilateral presentation. The term "leontiasis ossea" refers to the symmetrical and widespread involvement of the craniofacial bones by PFD-caused facial alterations resembling a "lion's face" [19].

Although, the risk of pathological fracture is highest in PFD, especially in McCune-Albright syndrome, de Mattos et al. reported that even 50% of MFD cases are prone to fracture [10]. In active or polyostotic lesions, the frequency of mutation may be higher compared to monostotic lesions. The FD involving the jaw shows symptoms ranging from painless to having dental malformations, malocclusion, and facial asymmetry, in addition to orbital dystopia, visual disturbance, hearing impairment, and nasal congestion [20].

FD is radiologically and histologically distinct from other bones, presumably as a result of its desmal origin [6]. Histopathological diagnosis of FD is particularly challenging because of the overlapping characteristics with other fibro-osseous tumours [6]. Three unique patterns can be seen at the microscopic examination: the hypercellular type, the Pagetoid type, and the conventional Chinese character shape [1]. It usually reveals foci of a little cellular fibrous connective tissue stroma with irregularly formed trabeculae of immature (woven) bone. Because of their curvilinear shape, the bony trabeculae resemble Chinese characters [21]. There is little to no osteoblastic rimming, and peri-trabecular clefting is prominent. In mature lesions, lamellar bone may be seen, without mitotic figures and pleomorphism [21]. The differential diagnosis of FD comprises simple bone cyst, non-ossifying fibroma, osseous-fibrous dysplasia, adamantinoma, low-grade intramedullary osteosarcoma, Paget’s disease, cemento-ossifying fibroma, florid cemento-osseous dysplasia, central giant cell granuloma, osteomyelitis [7]. Unlike neurofibromatosis, which exhibits smooth "coast of California" borders, FD is characterized by jagged "coast of Maine" borders. These lesions are typically located over the midline of the body, indicating variations in embryonic cell migration [22]. Several modalities have been employed to evaluate FD, including bone scintigraphy, CT, magnetic resonance imaging (MRI), and plain radiography. Bone biopsy is another diagnostic technique used to confirm FD diagnosis. The benefits and risks of a bone biopsy should be considered equally, as radiographic results, physical examination, and clinical history are often enough to provide a high-probability diagnosis [23]. Though CT imaging is necessary to ascertain the full degree of the lesion, panoramic radiography can be used as a crucial diagnostic technique [6]. Three types of FD can be identified by CT imaging: a cystic pattern (21%), a homogeneously dense pattern (23%), and a ground-glass appearance (56%) [24]. As the disease advances, it may have a uniform appearance or a lesion that is both radiopaque and radiolucent. Before ultimately becoming radiopaque, the radiographic variation first begins with radiolucency and transitions into a heterogeneous radiolucent and radiopaque component [6]. The malignant transformation of FD may be preceded by radiation, PFD, McCune-Albright syndrome, and growth hormone overexpression [25]. Accelerated expansion of the bone lesion and elevated alkaline phosphatase levels may serve as warning signs for a potential malignant transition [7]. Although fibro-osseous lesions are benign in nature and have a minimal incidence of malignant transformation, FD can transform into sarcomas, osteosarcomas, fibrosarcomas, chondrosarcomas, and angiosarcomas. Misdiagnosis is common due to its similarity to other fibro-osseous bone disorders, which might result in insufficient therapy. A basic understanding of the detection and management of FD is essential because this lesion, especially in the jaw, does not regress after adolescence [6].

Depending on the age of the patient, clinical management of FD might be very challenging. Observation, medication, and surgery are all part of the FD therapy regimen. FD lesions necessitate appropriate clinical surveillance due to their increased risk of pathologic fracture or deformity. Although there is not a specific drug that can change the course of the disease, medical care can be used for palliation, which includes the use of bisphosphonates, which have been shown to enhance function, reduce pain, and reduce the risk of fracture. Bisphosphonates are anti-resorptive agents that decrease bone turnover by inhibiting osteoclasts. In doing so, it is believed that bisphosphonates may be beneficial in the regeneration of bone for increasing bone mineral density (BMD) at lesion sites [26]. Surgery is performed for deformity repair, pathologic fracture prevention, and/or removal of bothersome lesions in individuals whose histopathology has been confirmed. Using an intraoral technique to remove damaged bone, conservative treatment is the standard of care management. Unlike cancellous bone grafts or bone graft substitutes, cortical bone grafts have an outstanding quality of rebuilt cortical bone [3].

## Conclusions

FD is a bone development disorder caused by a genetic abnormality that disrupts osteogenesis, resulting in the replacement of normal bone with an excess of fibrous tissue. It is a benign fibro-osseous disease that affects one or more bones. Development is slow and typically stops at puberty. In most cases FD affects children, but rarely adults. This condition has a broad clinical spectrum, ranging from insignificant solitary lesions to severe disease. It causes asymmetry, due to which facial aesthetics are compromised. Hence, bone remodelling must be performed according to age, gender, and the patient’s facial profile. A proper and regular follow-up is a must, to detect relapse or any malignant changes at an early stage.

## References

[1] Kim DY (2023). Current concepts of craniofacial fibrous dysplasia: pathophysiology and treatment. Arch Craniofac Surg.

[2] Sarangi S, Dutta S, Mitra S (2021). Fibrous dysplasia involving the left maxilla - report of a case with significant diagnostic aspects. Indian J Dent Adv.

[3] Kamble VR, Waghmare SN, Rangari AV, Meti M, Pohankar P, Paraye S (2021). Fibrous dysplasia of maxilla - a rare case report. Indian J Case Rep.

[4] Kim HY, Shim JH, Heo CY (2023). A rare skeletal disorder, fibrous dysplasia: a review of its pathogenesis and therapeutic prospects. Int J Mol Sci.

[5] Viganò L, Powier M, Viganò V, Casu C (2021). Fibrous dysplasia of the mandible: differential diagnosis. World Cancer Res J.

[6] Obermeier KT, Hartung JT, Hildebrandt T (2023). Fibrous dysplasia of the jaw: advances in imaging and treatment. J Clin Med.

[7] Berberi A, Aoun G, Khalaf E, Aad G (2021). Monostotic fibrous dysplasia of the mandible in a 9-year-old male patient treated with a conservative surgical treatment: a case report and 15-year follow-up. Case Rep Dent.

[8] Soluk-Tekkesin M, Sinanoglu A, Selvi F, Cakir Karabas H, Aksakalli N (2022). The importance of clinical and radiological findings for the definitive histopathologic diagnosis of benign fibro-osseous lesions of the jaws: study of 276 cases. J Stomatol Oral Maxillofac Surg.

[9] Aslan SG, Tezel K, Ordu-Gökkaya NK (2023). Fibrous dysplasia and McCune-Albright syndrome: a case report with review of literature on the rehabilitation approach. Turk J Phys Med Rehabil.

[10] MacDonald DS (2015). Maxillofacial fibro-osseous lesions. Clin Radiol.

[11] MacDonald-Jankowski DS (2004). Fibro-osseous lesions of the face and jaws. Clin Radiol.

[12] Idowu BD, Al-Adnani M, O'Donnell P (2007). A sensitive mutation-specific screening technique for GNAS1 mutations in cases of fibrous dysplasia: the first report of a codon 227 mutation in bone. Histopathology.

[13] Lumbroso S, Paris F, Sultan C (2002). McCune-Albright syndrome: molecular genetics. J Pediatr Endocrinol Metab.

[14] Weinstein LS, Yu S, Warner DR, Liu J (2001). Endocrine manifestations of stimulatory G protein alpha-subunit mutations and the role of genomic imprinting. Endocr Rev.

[15] Happle R (1986). The McCune-Albright syndrome: a lethal gene surviving by mosaicism. Clin Genet.

[16] Riminucci M, Collins MT, Fedarko NS (2003). FGF-23 in fibrous dysplasia of bone and its relationship to renal phosphate wasting. J Clin Invest.

[17] Riminucci M, Kuznetsov SA, Cherman N, Corsi A, Bianco P, Gehron Robey P (2003). Osteoclastogenesis in fibrous dysplasia of bone: in situ and in vitro analysis of IL-6 expression. Bone.

[18] Yang L, Wu H, Lu J, Teng L (2017). Prevalence of different forms and involved bones of craniofacial fibrous dysplasia. J Craniofac Surg.

[19] Byers PD, Jones AN (1969). Leontiasis ossea. Br J Surg.

[20] Chapurlat RD, Orcel P (2008). Fibrous dysplasia of bone and McCune-Albright syndrome. Best Pract Res Clin Rheumatol.

[21] Akinyamoju AO, Akinloye SJ, Okiti RO, Adeyemi BF (2024). Jaw fibro-osseous lesions: use of a predictive index in grading probable malignant changes and a review of cases. J Dent.

[22] Collins MT, Singer FR, Eugster E (2012). McCune-Albright syndrome and the extraskeletal manifestations of fibrous dysplasia. Orphanet J Rare Dis.

[23] Lee JS, FitzGibbon E, Butman JA (2002). Normal vision despite narrowing of the optic canal in fibrous dysplasia. N Engl J Med.

[24] Gupta D, Garg P, Mittal A (2017). Computed tomography in craniofacial fibrous dysplasia: a case series with review of literature and classification update. Open Dent J.

[25] Shi R, Li X, Zhang J, Chen F, Ma M, Feng Y, Li T (2022). Clinicopathological and genetic study of a rare occurrence: malignant transformation of fibrous dysplasia of the jaws. Mol Genet Genomic Med.

[26] DiCaprio MR, Enneking WF (2005). Fibrous dysplasia. Pathophysiology, evaluation, and treatment. J Bone Joint Surg Am.

